# (*E*)-5,6-Dimeth­oxy-2-(pyridin-4-yl­methyl­idene)-2,3-dihydro-1*H*-inden-1-one

**DOI:** 10.1107/S1600536810039486

**Published:** 2010-10-09

**Authors:** Mohamed Ashraf Ali, Rusli Ismail, Soo Choon Tan, Chin Sing Yeap, Hoong-Kun Fun

**Affiliations:** a Institute for Research in Molecular Medicine, Universiti Sains Malaysia, 11800 USM, Penang, Malaysia; b X-ray Crystallography Unit, School of Physics, Universiti Sains Malaysia, 11800 USM, Penang, Malaysia

## Abstract

The mol­ecule of the title compound, C_17_H_15_NO_3_, is slightly twisted, with a dihedral angle of 12.12 (3)° between the dihydro­indenone group and the pyridine ring. In the crystal, mol­ecules are connected into layers parallel to the *ab* plane *via* inter­molecular C—H⋯O hydrogen bonds. Weak π–π [centroid–centroid distance = 3.5680 (6) Å] inter­actions are also observed.

## Related literature

For general background and the biological activity of chalcone derivatives, see: Nowakowska (2008 ▶); Akihisa *et al.* (2006 ▶); Narender *et al.* (2005 ▶); Zhang *et al.* (2006 ▶); Dicarlo *et al.* (1999 ▶); Heidenreich *et al.* (2008 ▶); Syed *et al.* (2008 ▶); D’Archivio *et al.* (2008 ▶). For a related structure, see: Ali *et al.* (2010 ▶). For the stability of the temperature controller used in the data collection, see: Cosier & Glazer (1986 ▶).
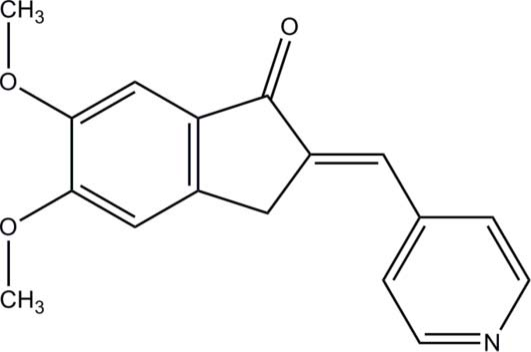


## Experimental

### 

#### Crystal data

C_17_H_15_NO_3_
*M* *_r_* = 281.30
Monoclinic, 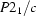

*a* = 10.7572 (14) Å
*b* = 8.6057 (11) Å
*c* = 17.2961 (17) Å
β = 123.394 (6)°
*V* = 1336.8 (3) Å^3^
*Z* = 4
Mo *K*α radiation
μ = 0.10 mm^−1^
*T* = 100 K
0.45 × 0.32 × 0.23 mm

#### Data collection

Bruker APEXII Duo CCD area-detector diffractometer
Absorption correction: multi-scan (*SADABS*; Bruker, 2009 ▶) *T*_min_ = 0.958, *T*_max_ = 0.979
21745 measured reflections
5874 independent reflections
5138 reflections with *I* > 2σ(*I*)
*R*_int_ = 0.023

#### Refinement

*R*[*F* ^2^ > 2σ(*F* ^2^)] = 0.039
*wR*(*F* ^2^) = 0.132
*S* = 1.10
5874 reflections
192 parameters
H-atom parameters constrained
Δρ_max_ = 0.61 e Å^−3^
Δρ_min_ = −0.27 e Å^−3^

### 

Data collection: *APEX2* (Bruker, 2009 ▶); cell refinement: *SAINT* (Bruker, 2009 ▶); data reduction: *SAINT*; program(s) used to solve structure: *SHELXTL* (Sheldrick, 2008 ▶); program(s) used to refine structure: *SHELXTL*; molecular graphics: *SHELXTL*; software used to prepare material for publication: *SHELXTL* and *PLATON* (Spek, 2009 ▶).

## Supplementary Material

Crystal structure: contains datablocks global, I. DOI: 10.1107/S1600536810039486/rz2492sup1.cif

Structure factors: contains datablocks I. DOI: 10.1107/S1600536810039486/rz2492Isup2.hkl

Additional supplementary materials:  crystallographic information; 3D view; checkCIF report

## Figures and Tables

**Table 1:** Hydrogen-bond geometry (Å, °)

*D*—H⋯*A*	*D*—H	H⋯*A*	*D*⋯*A*	*D*—H⋯*A*
C14—H14*A*⋯O2^i^	0.93	2.57	3.4787 (12)	167
C14—H14*A*⋯O3^i^	0.93	2.57	3.2708 (12)	133
C16—H16*A*⋯O1^ii^	0.96	2.53	3.0486 (11)	114

Symmetry codes: (i) 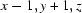; (ii) 
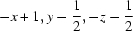
.

**Fig. 1.**
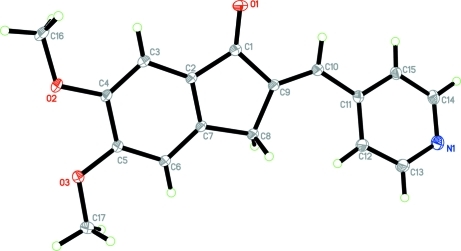
The molecular structure of the title compound with atom labels and 50% probability ellipsoids for non-H atoms.

**Fig. 2.**
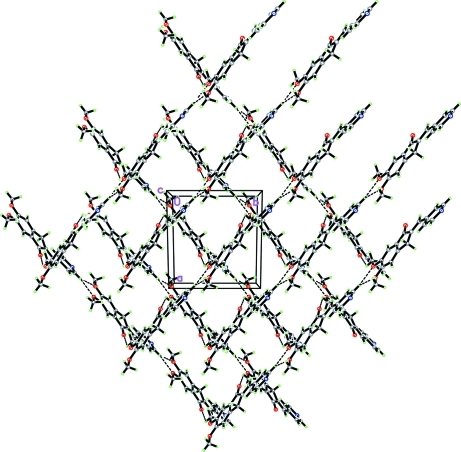
The crystal packing of title compound, showing chains arranged anti-parallelly. Intermolecular hydrogen bonds are shown as dashed lines.

**Fig. 3.**
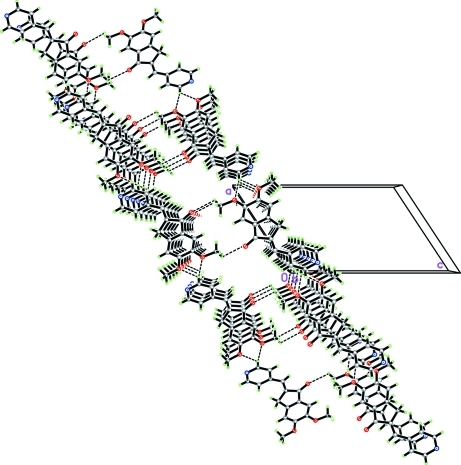
The crystal packing of title compound, showing a two-dimensional plane parallel to ab plane. Intermolecular hydrogen bonds are shown as dashed lines.

## supplementary crystallographic information

### Comment

Chalcone moieties are common substructures in numerous natural products belonging to the flavonoid family (Nowakowska, 2008; Akihisa *et al.* 2006; Narender *et al.*, 2005; Zhang *et al.*, 2006).Chalcone derivatives are very versatile as physiologically active compounds and substrates for the evaluation of various organic syntheses. Chalcones, one of the major classes of natural products with widespread distribution in spices, tea, beer, fruits and vegetables, have been recently subject of great interest for their pharmacological activities (Dicarlo *et al.*, 2009). Prostate cancer is one of the most commonly diagnosed cancers in men, and the second leading cause of cancer deaths in the European Union and United States of America (Heidenreich *et al.*, 2008). Many antitumor drugs have been developed for prostate cancer patients, but their intolerable systemic toxicity often limits their clinical use. Chemoprevention is one of the most promising approaches in prostate cancer research, in which natural or synthetic agents are used to prevent this malignant disease (Heidenreich *et al.*, 2008; Syed *et al.*, 2008; D'Archivio *et al.*, 2008).

The molecular structure of the title compound is slightly twisted (Fig. 1). The torsion angles of the two methoxy groups are [C16–O2–C4–C5] -175.13 (6) and [C17–O3–C5–C4] -178.48 (6)°. The maximum deviation of the dihydroindenone group is 0.028 (1) Å and it makes dihedral angle of 12.12 (3)° with the pyridine ring [C11–C13/N1/C14–C15]. The geometry parameters are comparable to those observed in a closely related structure (Ali *et al.*, 2010).

In the crystal structure, the molecules are linked together into chains by a bifurcated hydrogen bonds involving the intermolecular C14—H14A···O3 and C14—H14A···O3 hydrogen bonds (Table 1) generating a *R*^2^_1_(5) ring motif. These chains are arranged in an anti-parallel layer (Fig. 2) and each pair of anti-parallel layers are interconnected into a two-dimensional plane parallel to *ab* plane *via* intermolecular C16—H16A···O1 hydrogen bonds (Fig. 3, Table 1). Weak π···π interactions are also observed [*Cg*1···*Cg*2^iii^ of 3.5680 (6) Å; (iii) 1 - *x*, 1 - *y*, -*z. Cg*1 and *Cg*2 are centroids of C1–C2/C7–C9 and C2–C7 ring, respectively].

### Experimental

A mixture of 5,6-dimethoxy-2,3-dihydro-1*H*-indene-1-one (0.001 mmol) and isonicotinaldehyde (0.001 mmol) were dissolved in methanol (10 ml) and 30% sodium hydroxide solution (5 ml) was added and the solution stirred for 5 h. After completion of the reaction as evident from TLC, the mixture was poured into crushed ice then neutralized with concentrated HCl. The precipitated solid was filtered, washed with water and recrystallized from ethanol to reveal the title compound as light yellow crystals.

### Refinement

All hydrogen atoms were positioned geometrically [C–H = 0.93–0.97 Å] and refined using a riding model with *U*_iso_(H) = 1.2 *U*_eq_(C) or 1.5 *U*_eq_(C) for methyl H atoms. A rotating-group model was applied for the methyl groups.

### Crystal data

**Table:**

C_17_H_15_NO_3_	*F*(000) = 592
*M**_r_* = 281.30	*D*_x_ = 1.398 Mg m^−^^3^
Monoclinic, *P*2_1_/*c*	Mo *K*α radiation, λ = 0.71073 Å
Hall symbol: -P 2ybc	Cell parameters from 9933 reflections
*a* = 10.7572 (14) Å	θ = 2.8–35.1°
*b* = 8.6057 (11) Å	µ = 0.10 mm^−^^1^
*c* = 17.2961 (17) Å	*T* = 100 K
β = 123.394 (6)°	Block, yellow
*V* = 1336.8 (3) Å^3^	0.45 × 0.32 × 0.23 mm
*Z* = 4	

### Data collection

**Table:**

Bruker APEXII Duo CCD area-detector diffractometer	5874 independent reflections
Radiation source: fine-focus sealed tube	5138 reflections with *I* > 2σ(*I*)
graphite	*R*_int_ = 0.023
φ and ω scans	θ_max_ = 35.1°, θ_min_ = 2.8°
Absorption correction: multi-scan (*SADABS*; Bruker, 2009)	*h* = −17→17
*T*_min_ = 0.958, *T*_max_ = 0.979	*k* = −13→13
21745 measured reflections	*l* = −17→27

### Refinement

**Table:**

Refinement on *F*^2^	Primary atom site location: structure-invariant direct methods
Least-squares matrix: full	Secondary atom site location: difference Fourier map
*R*[*F*^2^ > 2σ(*F*^2^)] = 0.039	Hydrogen site location: inferred from neighbouring sites
*wR*(*F*^2^) = 0.132	H-atom parameters constrained
*S* = 1.10	*w* = 1/[σ^2^(*F*_o_^2^) + (0.0783*P*)^2^ + 0.2242*P*] where *P* = (*F*_o_^2^ + 2*F*_c_^2^)/3
5874 reflections	(Δ/σ)_max_ < 0.001
192 parameters	Δρ_max_ = 0.61 e Å^−^^3^
0 restraints	Δρ_min_ = −0.26 e Å^−^^3^

### Special details

**Table:**

Experimental. The crystal was placed in the cold stream of an Oxford Cryosystems Cobra open-flow nitrogen cryostat (Cosier & Glazer, 1986) operating at 100.0 (1) K.
Geometry. All e.s.d.'s (except the e.s.d. in the dihedral angle between two l.s. planes) are estimated using the full covariance matrix. The cell e.s.d.'s are taken into account individually in the estimation of e.s.d.'s in distances, angles and torsion angles; correlations between e.s.d.'s in cell parameters are only used when they are defined by crystal symmetry. An approximate (isotropic) treatment of cell e.s.d.'s is used for estimating e.s.d.'s involving l.s. planes.
Refinement. Refinement of *F*^2^ against ALL reflections. The weighted *R*-factor *wR* and goodness of fit *S* are based on *F*^2^, conventional *R*-factors *R* are based on *F*, with *F* set to zero for negative *F*^2^. The threshold expression of *F*^2^ > σ(*F*^2^) is used only for calculating *R*-factors(gt) *etc*. and is not relevant to the choice of reflections for refinement. *R*-factors based on *F*^2^ are statistically about twice as large as those based on *F*, and *R*- factors based on ALL data will be even larger.

### Fractional atomic coordinates and isotropic or equivalent isotropic displacement parameters (Å^2^)

**Table:**

	*x*	*y*	*z*	*U*_iso_*/*U*_eq_	
O1	0.32457 (7)	0.81081 (8)	−0.16256 (4)	0.02006 (13)	
O2	0.80183 (6)	0.43182 (7)	−0.05682 (4)	0.01665 (11)	
O3	0.92680 (6)	0.42839 (7)	0.11848 (4)	0.01546 (11)	
N1	0.16589 (8)	1.13656 (9)	0.15678 (5)	0.01914 (13)	
C1	0.41258 (8)	0.78448 (8)	−0.07968 (5)	0.01273 (12)	
C2	0.54736 (7)	0.68903 (7)	−0.03693 (4)	0.01082 (11)	
C3	0.60522 (8)	0.60592 (8)	−0.08026 (4)	0.01211 (12)	
H3A	0.5587	0.6093	−0.1442	0.015*	
C4	0.73270 (7)	0.51920 (8)	−0.02574 (4)	0.01175 (12)	
C5	0.80361 (7)	0.51765 (8)	0.07250 (4)	0.01137 (11)	
C6	0.74738 (7)	0.60432 (8)	0.11466 (4)	0.01135 (11)	
H6A	0.7955	0.6055	0.1787	0.014*	
C7	0.61696 (7)	0.68941 (7)	0.05839 (4)	0.01039 (11)	
C8	0.53304 (8)	0.78599 (8)	0.08831 (4)	0.01196 (12)	
H8A	0.5018	0.7233	0.1213	0.014*	
H8B	0.5934	0.8717	0.1275	0.014*	
C9	0.40070 (7)	0.84391 (8)	−0.00227 (4)	0.01164 (11)	
C10	0.28495 (8)	0.93423 (8)	−0.02118 (5)	0.01312 (12)	
H10A	0.2176	0.9567	−0.0835	0.016*	
C11	0.24992 (7)	1.00219 (8)	0.04230 (5)	0.01233 (12)	
C12	0.31954 (9)	0.96061 (10)	0.13540 (5)	0.01933 (15)	
H12A	0.3965	0.8883	0.1618	0.023*	
C13	0.27285 (10)	1.02821 (11)	0.18809 (6)	0.02310 (17)	
H13A	0.3188	0.9962	0.2493	0.028*	
C14	0.10022 (8)	1.17804 (9)	0.06768 (5)	0.01567 (13)	
H14A	0.0262	1.2536	0.0440	0.019*	
C15	0.13705 (8)	1.11388 (8)	0.00888 (5)	0.01385 (12)	
H15A	0.0866	1.1453	−0.0527	0.017*	
C16	0.74321 (9)	0.43840 (10)	−0.15362 (5)	0.01808 (14)	
H16A	0.8014	0.3730	−0.1670	0.027*	
H16B	0.7471	0.5435	−0.1708	0.027*	
H16C	0.6418	0.4032	−0.1881	0.027*	
C17	1.00081 (9)	0.41893 (10)	0.21693 (5)	0.01939 (14)	
H17A	1.0849	0.3505	0.2415	0.029*	
H17B	0.9330	0.3794	0.2319	0.029*	
H17C	1.0342	0.5205	0.2434	0.029*	

### Atomic displacement parameters (Å^2^)

**Table:**

	*U*^11^	*U*^22^	*U*^33^	*U*^12^	*U*^13^	*U*^23^
O1	0.0204 (3)	0.0275 (3)	0.0098 (2)	0.0087 (2)	0.0067 (2)	0.00324 (19)
O2	0.0182 (2)	0.0220 (3)	0.0120 (2)	0.00551 (19)	0.00975 (19)	−0.00182 (18)
O3	0.0142 (2)	0.0200 (2)	0.0111 (2)	0.00625 (18)	0.00627 (18)	0.00077 (17)
N1	0.0189 (3)	0.0226 (3)	0.0175 (3)	0.0056 (2)	0.0109 (2)	−0.0015 (2)
C1	0.0138 (3)	0.0142 (3)	0.0106 (3)	0.0021 (2)	0.0070 (2)	0.0008 (2)
C2	0.0119 (2)	0.0118 (2)	0.0096 (2)	0.00066 (19)	0.0065 (2)	−0.00052 (19)
C3	0.0132 (3)	0.0140 (3)	0.0095 (2)	0.0003 (2)	0.0065 (2)	−0.00105 (19)
C4	0.0130 (3)	0.0135 (3)	0.0106 (2)	0.0005 (2)	0.0076 (2)	−0.00177 (19)
C5	0.0115 (2)	0.0128 (3)	0.0104 (2)	0.00140 (19)	0.0064 (2)	−0.00031 (19)
C6	0.0122 (2)	0.0129 (3)	0.0095 (2)	0.00100 (19)	0.0063 (2)	−0.00042 (19)
C7	0.0118 (2)	0.0111 (2)	0.0094 (2)	0.00076 (19)	0.0066 (2)	−0.00013 (18)
C8	0.0136 (3)	0.0131 (3)	0.0101 (2)	0.0024 (2)	0.0071 (2)	0.00030 (19)
C9	0.0131 (3)	0.0122 (3)	0.0105 (2)	0.0015 (2)	0.0071 (2)	0.00038 (19)
C10	0.0138 (3)	0.0144 (3)	0.0115 (3)	0.0024 (2)	0.0072 (2)	0.0007 (2)
C11	0.0121 (3)	0.0128 (3)	0.0129 (3)	0.0015 (2)	0.0074 (2)	−0.0003 (2)
C12	0.0212 (3)	0.0241 (3)	0.0141 (3)	0.0106 (3)	0.0105 (3)	0.0025 (2)
C13	0.0249 (4)	0.0309 (4)	0.0149 (3)	0.0132 (3)	0.0118 (3)	0.0027 (3)
C14	0.0146 (3)	0.0147 (3)	0.0185 (3)	0.0023 (2)	0.0096 (3)	−0.0008 (2)
C15	0.0131 (3)	0.0139 (3)	0.0154 (3)	0.0019 (2)	0.0083 (2)	0.0009 (2)
C16	0.0212 (3)	0.0235 (3)	0.0127 (3)	0.0006 (3)	0.0113 (3)	−0.0035 (2)
C17	0.0184 (3)	0.0259 (4)	0.0118 (3)	0.0083 (3)	0.0070 (2)	0.0035 (2)

### Geometric parameters (Å, °)

**Table:**

O1—C1	1.2283 (8)	C8—H8A	0.9700
O2—C4	1.3593 (8)	C8—H8B	0.9700
O2—C16	1.4289 (9)	C9—C10	1.3465 (9)
O3—C5	1.3486 (8)	C10—C11	1.4646 (10)
O3—C17	1.4310 (9)	C10—H10A	0.9300
N1—C13	1.3419 (10)	C11—C12	1.3980 (10)
N1—C14	1.3427 (10)	C11—C15	1.3999 (10)
C1—C2	1.4640 (10)	C12—C13	1.3874 (11)
C1—C9	1.5029 (9)	C12—H12A	0.9300
C2—C7	1.3857 (9)	C13—H13A	0.9300
C2—C3	1.4050 (9)	C14—C15	1.3928 (10)
C3—C4	1.3796 (9)	C14—H14A	0.9300
C3—H3A	0.9300	C15—H15A	0.9300
C4—C5	1.4291 (9)	C16—H16A	0.9600
C5—C6	1.3926 (9)	C16—H16B	0.9600
C6—C7	1.3959 (9)	C16—H16C	0.9600
C6—H6A	0.9300	C17—H17A	0.9600
C7—C8	1.5124 (9)	C17—H17B	0.9600
C8—C9	1.5081 (9)	C17—H17C	0.9600
			
C4—O2—C16	117.18 (6)	C1—C9—C8	108.57 (5)
C5—O3—C17	117.17 (6)	C9—C10—C11	129.30 (6)
C13—N1—C14	116.14 (7)	C9—C10—H10A	115.3
O1—C1—C2	127.28 (6)	C11—C10—H10A	115.3
O1—C1—C9	126.02 (6)	C12—C11—C15	116.38 (6)
C2—C1—C9	106.69 (5)	C12—C11—C10	124.44 (6)
C7—C2—C3	121.87 (6)	C15—C11—C10	119.16 (6)
C7—C2—C1	109.63 (5)	C13—C12—C11	119.41 (7)
C3—C2—C1	128.51 (6)	C13—C12—H12A	120.3
C4—C3—C2	118.44 (6)	C11—C12—H12A	120.3
C4—C3—H3A	120.8	N1—C13—C12	124.49 (7)
C2—C3—H3A	120.8	N1—C13—H13A	117.8
O2—C4—C3	125.68 (6)	C12—C13—H13A	117.8
O2—C4—C5	114.52 (6)	N1—C14—C15	123.45 (7)
C3—C4—C5	119.80 (6)	N1—C14—H14A	118.3
O3—C5—C6	124.43 (6)	C15—C14—H14A	118.3
O3—C5—C4	114.50 (6)	C14—C15—C11	120.09 (7)
C6—C5—C4	121.07 (6)	C14—C15—H15A	120.0
C5—C6—C7	118.39 (6)	C11—C15—H15A	120.0
C5—C6—H6A	120.8	O2—C16—H16A	109.5
C7—C6—H6A	120.8	O2—C16—H16B	109.5
C2—C7—C6	120.38 (6)	H16A—C16—H16B	109.5
C2—C7—C8	112.02 (6)	O2—C16—H16C	109.5
C6—C7—C8	127.58 (6)	H16A—C16—H16C	109.5
C9—C8—C7	103.07 (5)	H16B—C16—H16C	109.5
C9—C8—H8A	111.1	O3—C17—H17A	109.5
C7—C8—H8A	111.1	O3—C17—H17B	109.5
C9—C8—H8B	111.1	H17A—C17—H17B	109.5
C7—C8—H8B	111.1	O3—C17—H17C	109.5
H8A—C8—H8B	109.1	H17A—C17—H17C	109.5
C10—C9—C1	120.04 (6)	H17B—C17—H17C	109.5
C10—C9—C8	131.39 (6)		
			
O1—C1—C2—C7	179.48 (7)	C5—C6—C7—C2	−1.08 (10)
C9—C1—C2—C7	−1.11 (8)	C5—C6—C7—C8	177.43 (6)
O1—C1—C2—C3	−0.64 (12)	C2—C7—C8—C9	0.57 (7)
C9—C1—C2—C3	178.78 (7)	C6—C7—C8—C9	−178.05 (7)
C7—C2—C3—C4	1.86 (10)	O1—C1—C9—C10	0.39 (12)
C1—C2—C3—C4	−178.02 (7)	C2—C1—C9—C10	−179.04 (6)
C16—O2—C4—C3	4.42 (10)	O1—C1—C9—C8	−179.11 (7)
C16—O2—C4—C5	−175.13 (6)	C2—C1—C9—C8	1.46 (7)
C2—C3—C4—O2	179.51 (6)	C7—C8—C9—C10	179.35 (7)
C2—C3—C4—C5	−0.96 (10)	C7—C8—C9—C1	−1.23 (7)
C17—O3—C5—C6	2.22 (10)	C1—C9—C10—C11	178.89 (7)
C17—O3—C5—C4	−178.48 (6)	C8—C9—C10—C11	−1.74 (13)
O2—C4—C5—O3	−0.69 (9)	C9—C10—C11—C12	−12.38 (13)
C3—C4—C5—O3	179.73 (6)	C9—C10—C11—C15	169.28 (7)
O2—C4—C5—C6	178.64 (6)	C15—C11—C12—C13	1.08 (12)
C3—C4—C5—C6	−0.94 (10)	C10—C11—C12—C13	−177.30 (8)
O3—C5—C6—C7	−178.78 (6)	C14—N1—C13—C12	1.08 (14)
C4—C5—C6—C7	1.96 (10)	C11—C12—C13—N1	−1.95 (15)
C3—C2—C7—C6	−0.83 (10)	C13—N1—C14—C15	0.58 (12)
C1—C2—C7—C6	179.06 (6)	N1—C14—C15—C11	−1.33 (12)
C3—C2—C7—C8	−179.56 (6)	C12—C11—C15—C14	0.43 (11)
C1—C2—C7—C8	0.33 (8)	C10—C11—C15—C14	178.90 (6)

### Hydrogen-bond geometry (Å, °)

**Table:**

*D*—H···*A*	*D*—H	H···*A*	*D*···*A*	*D*—H···*A*
C14—H14A···O2^i^	0.93	2.57	3.4787 (12)	167
C14—H14A···O3^i^	0.93	2.57	3.2708 (12)	133
C16—H16A···O1^ii^	0.96	2.53	3.0486 (11)	114

Symmetry codes: (i) *x*−1, *y*+1, *z*; (ii) −*x*+1, *y*−1/2, −*z*−1/2.
